# A War Emergency Committee for London

**Published:** 1915-05-22

**Authors:** 


### A WAR EMERGENCY COMMITTEE FOR LONDON.

The title is an alarming one, but the thing itself is not so serious as its name suggests. First, the Committee is concerned, not with combatant emergencies, but with the supply of doctors. Secondly, it is composed of quiet citizens whose most warlike moments are found in the agitations which disturb the domestic peace of the British Medical Association; and, thirdly, though it has been in existence for some weeks nothing terrible has happened. There is, therefore, no need for anxiety in the intimation that the Metropolitan Counties Branch of the B.M.A. has appointed a War Emergency Committee. We make no pretence to know the exact terms of the reference with which the Committee is charged, but from a circular which has been issued by it we gather that its purpose is to try, so far as the Metropolis is concerned, to organise a helpful reply to the appeal made by the Army medical authorities.

Perhaps we may be permitted to express our sympathy with this aim and, so far as we know them, with the methods of procedure which the Committee has adopted. Certainly what is wanted in the first instance is confident and exact information. How many practitioners are there under forty years of age able and willing to take commissions in the Army and to accompany the troops which are to be sent abroad? The Committee, by means of its circular-letter, ought to get an answer to this question, and we hope it will get also—what is of equal importance—the reasons why many men who are perfectly willing to join the Army feel themselves unable to do so. It is only necessary to read the medical journals to discover that there are more difficulties than the authorities realise. The question of finance is a very serious one. At least it is very serious in the case of men who are married and settled in practice. To consider the future most carefully is incumbent on these, and an indefinite and possibly prolonged absence from their work may readily mean that on their return there is little work left for them. In spite of the good will and loyalty of immediate neighbours patients drift away, and this especially in large towns where united and homogeneous action of the profession is hardly possible. The present offer is of a gratuity of £60 at the end of the war. There are many suggestions that such a sum is utterly inadequate to meet the position of men situated as just described. Another point which is being discussed is the indefinite term of service. If a period of time could be fixed, say six months or twelve months, a practitioner might be able to make definite arrangements for his absence and could reasonably hope on his return to find his practice substantially intact. But a vague term such as "the period of the war" makes the provision of a supply difficult and introduces all round a sense of helplessness and uncertainty. How far these two influences, the money element and the time element, are operative we do not at present know, but we hope that ere long the work of the Committee will provide us with the figures. Then the authorities will understand what conditions are really necessary in order to obtain the assistance they require. What they propose just now is largely a shot in the dark, for they have no experience to enable them to appreciate the conditions of civil practice.

We are glad that the Committee is not limiting its investigations to the younger men. The seniors, though the heroic post is denied them, must equally fall in on the line of duty. Part-time service in the military hospitals, medical work in camps, examination of recruits, and so on, are all duties which will afford them an opportunity to help. When the numbers available are known, then will be the chance of distributing the work. We hope the Committee will set itself to gain some exact information regarding the arrangements in our hospitals and other institutions. There is a real danger that in these may be detained a number of young men who belong to the class specially wanted with the new Armies. That some must stay at home may perhaps be allowed. But let there be no more than is absolutely necessary. There are various sources from which house physicians and house surgeons can be obtained other than young men who have recently taken their diplomas. Pressure necessary to enforce this view must be steadily applied to the visiting staffs of our hospitals. If they will give more personal attendance to the wards fewer resident officers will be required. Here is their opportunity for service and sacrifice, and we suggest that the new Committee should keep this opportunity prominently before their eyes. Its aim deserves cordial recognition, and we trust it will find a successful mission.

